# Supplementary figures and images for: Dissolved greenhouse gases and benthic microbial communities in coastal wetlands of the Chilean coast semiarid region
Source: PLoS One. 2022 Sep 29;17(9):e0271208. doi: 10.1371/journal.pone.0271208 (PMC9522034; doi:10.1371/journal.pone.0271208)

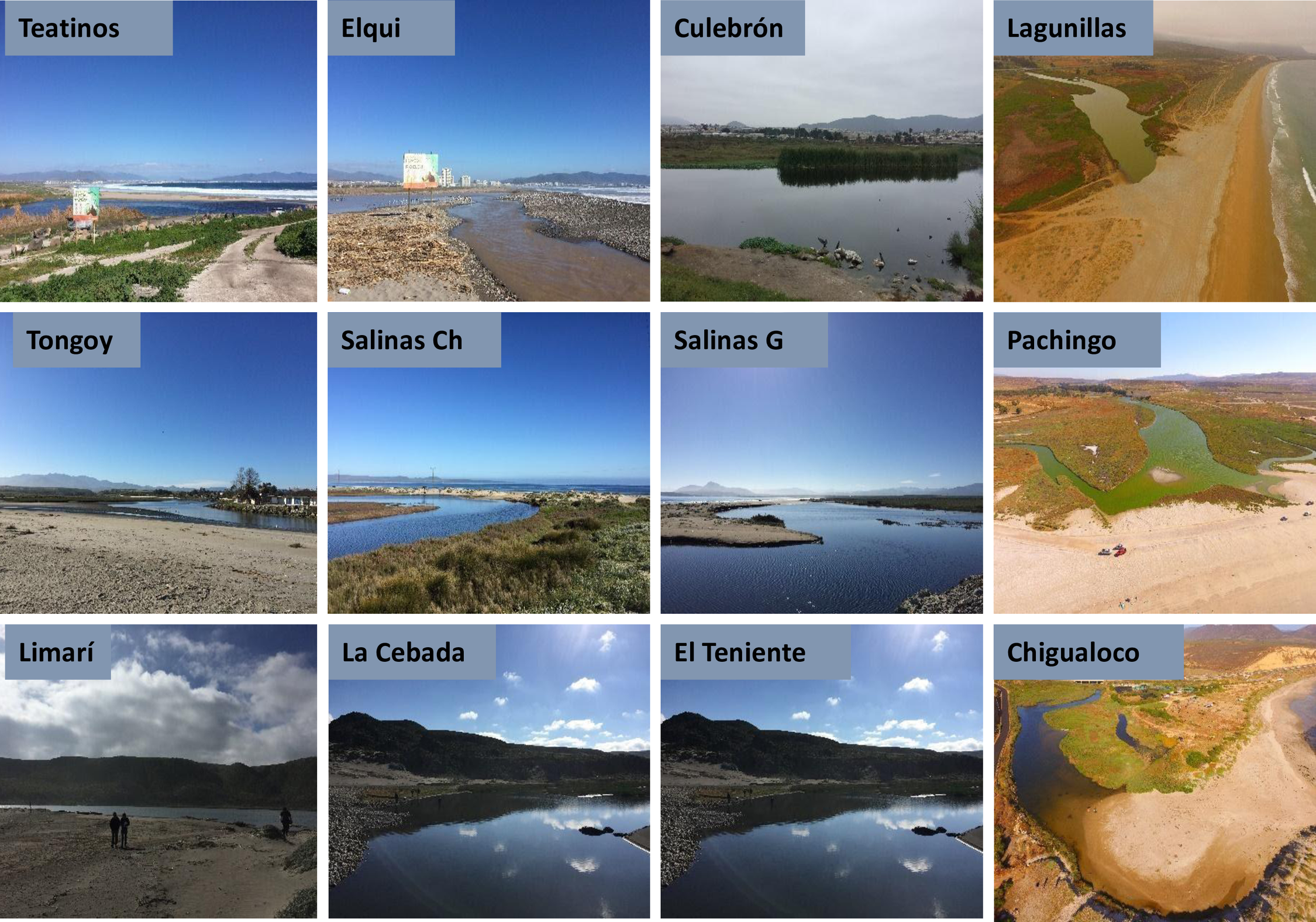

Supplement: S1 Fig — (TIF) [file pone.0271208.s008.tif]

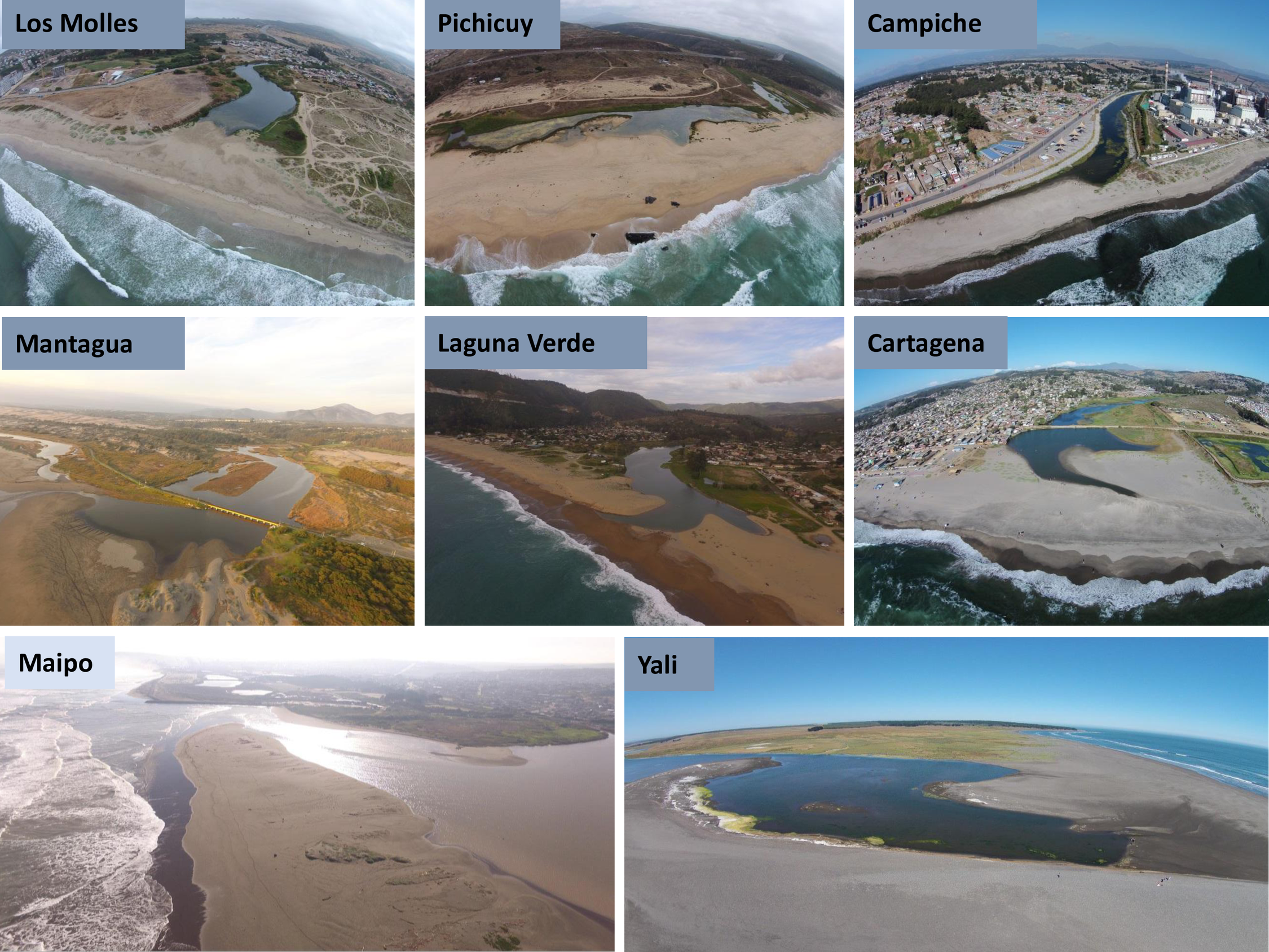

Supplement: S2 Fig — (TIF) [file pone.0271208.s009.tif]

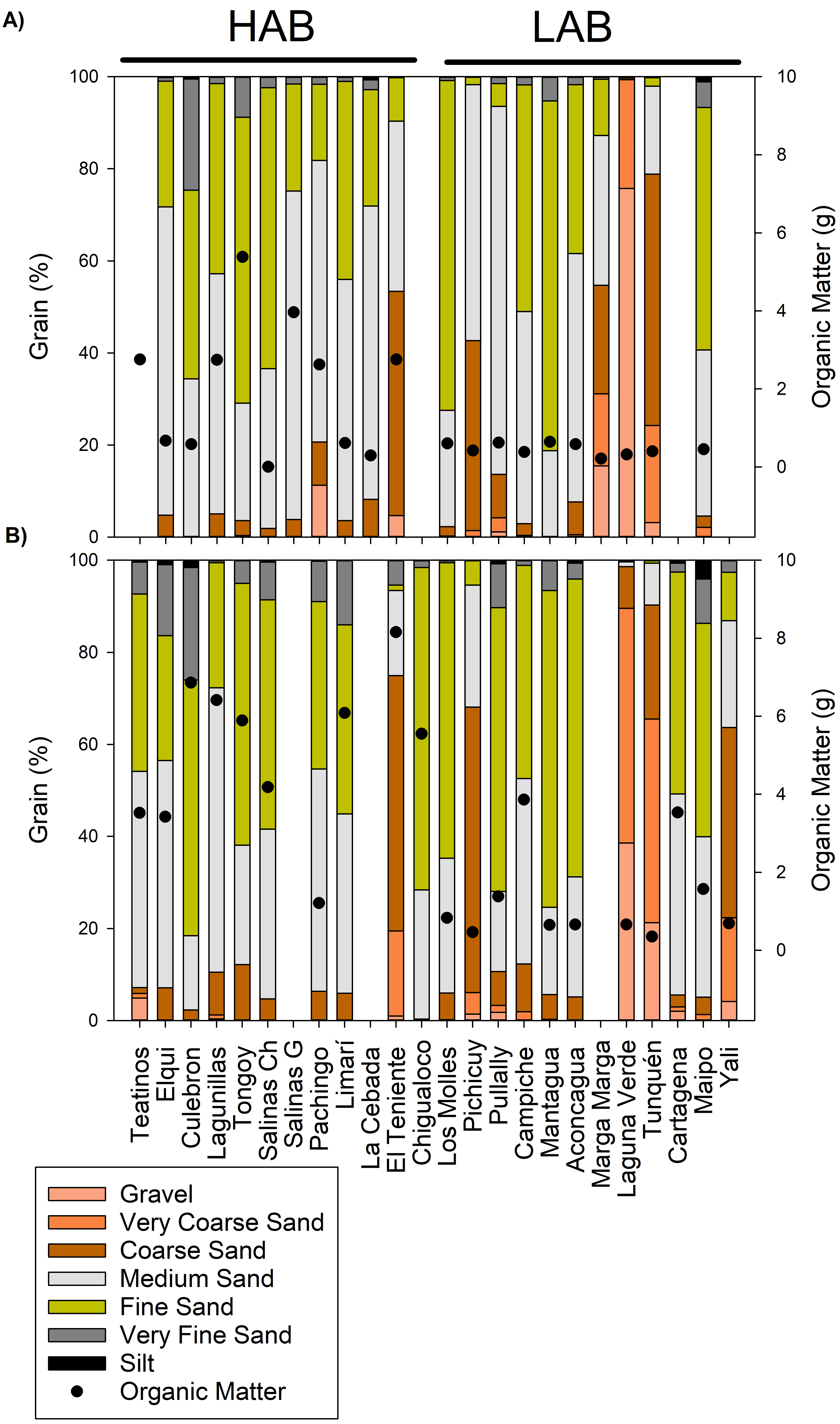

Supplement: S3 Fig — Granulometry and organic matter in the sediments of the sampled wetlands in winter (A) and summer (B). Teatinos, Chigualoco, Cartagena and Yali wetlands were only measured in summer and Salinas G, La Cebada and Marga Marga wetlands were only measured in winter. (TIF) [file pone.0271208.s010.tif]

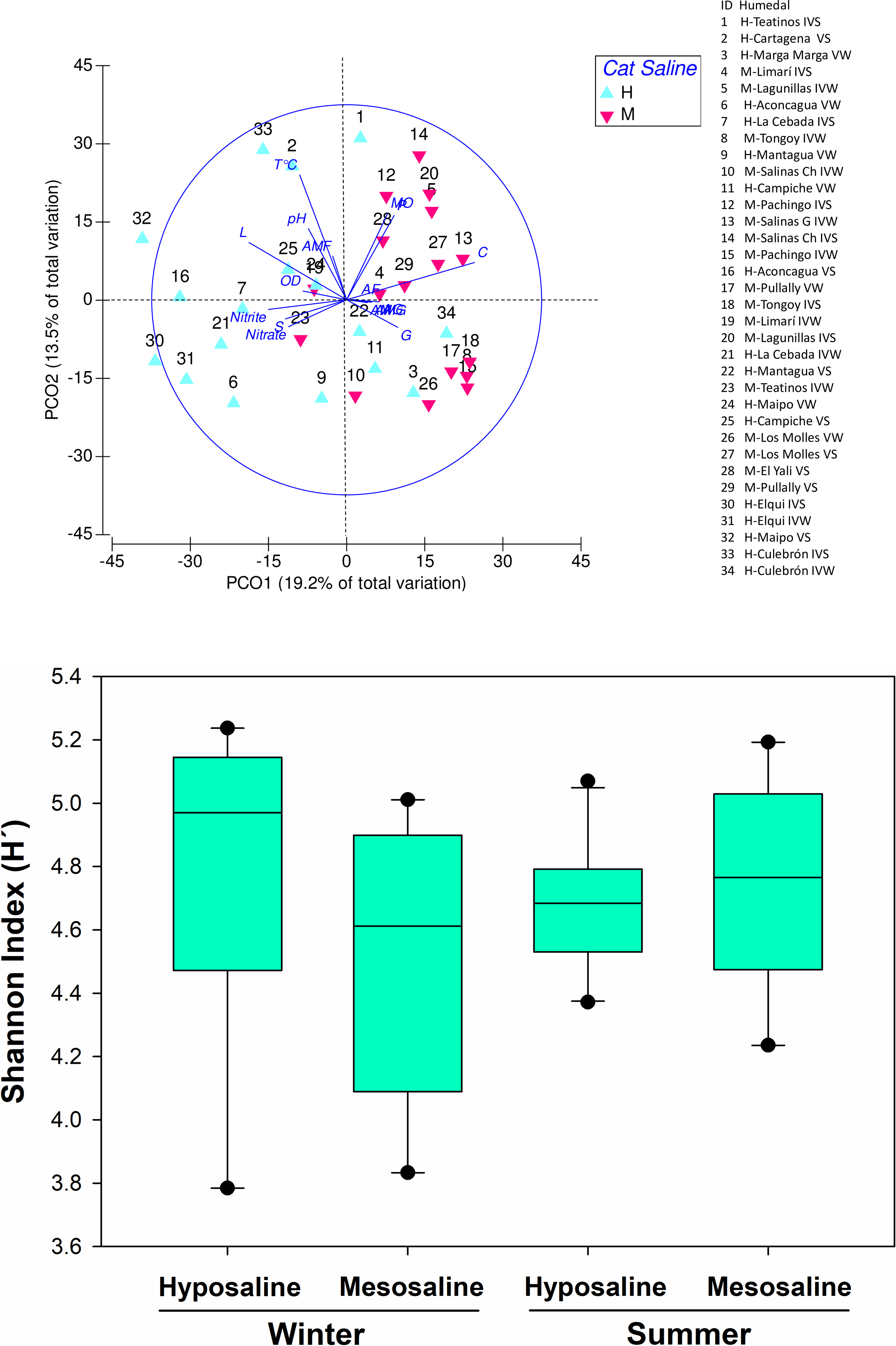

Supplement: S4 Fig — A) Principal Coordinate Analysis in hyposaline and mesosaline wetlands. Acronyms in the wetland names are H = hyposaline, M = mesosaline, IV = Coquimbo Region, V = Valparaíso Region, W = Winter, V = Summer. B) Boxplot (median, quartiles, and outliers) showing grouped richness values considering the saline categories. (TIF) [file pone.0271208.s011.tif]

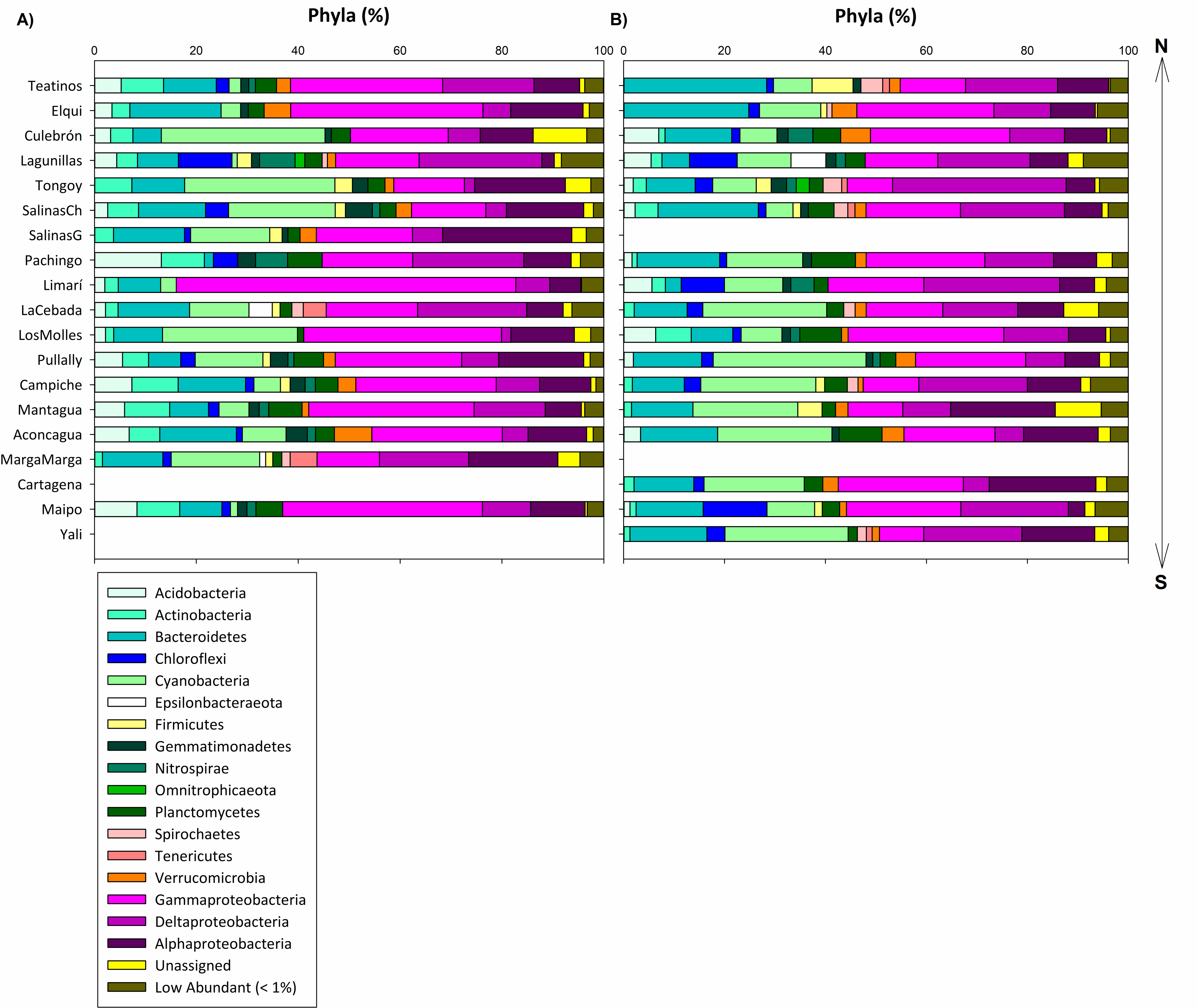

Supplement: S5 Fig — Benthic bacterial phyla observed in winter (A) and summer (B). The Proteobacteria phylum was separated in the Gamma, Delta and Alphaproteobacteria classes. Low abundant Phyla (<0.1%, Acetothermia, Aegiribacteria, AncK6, Armatimonadetes, Atribacteria, BHI80-139, BRC1, CK-2C2-2, Caldiserica, Calditrichaeota, Chlamydiae, Cloacimonetes, Coprothermobacteraeota, Dadabacteria, Deferribacteres, Deinococcus-Thermus, Dependentiae, Elusimicrobia, Entotheonellaeota, Epsilonbacteraeota, FBP, FCPU426, Fibrobacteres, Fusobacteria, GAL15, Halanaerobiaeota, Hydrogenedentes, Kiritimatiellaeota, LCP-89, Latescibacteria, Lentisphaerae, MAT-CR-M4-B07, Margulisbacteria, Marinimicrobia (SAR406 clade), Modulibacteria, Nitrospinae, Omnitrophicaeota, PAUC34f, Patescibacteria, Poribacteria, Rokubacteria, Schekmanbacteria, Synergistetes, TA06, Thermotogae, WOR-1, WPS-2, WS1, WS2, WS4, Zixibacteria. (TIF) [file pone.0271208.s012.tif]

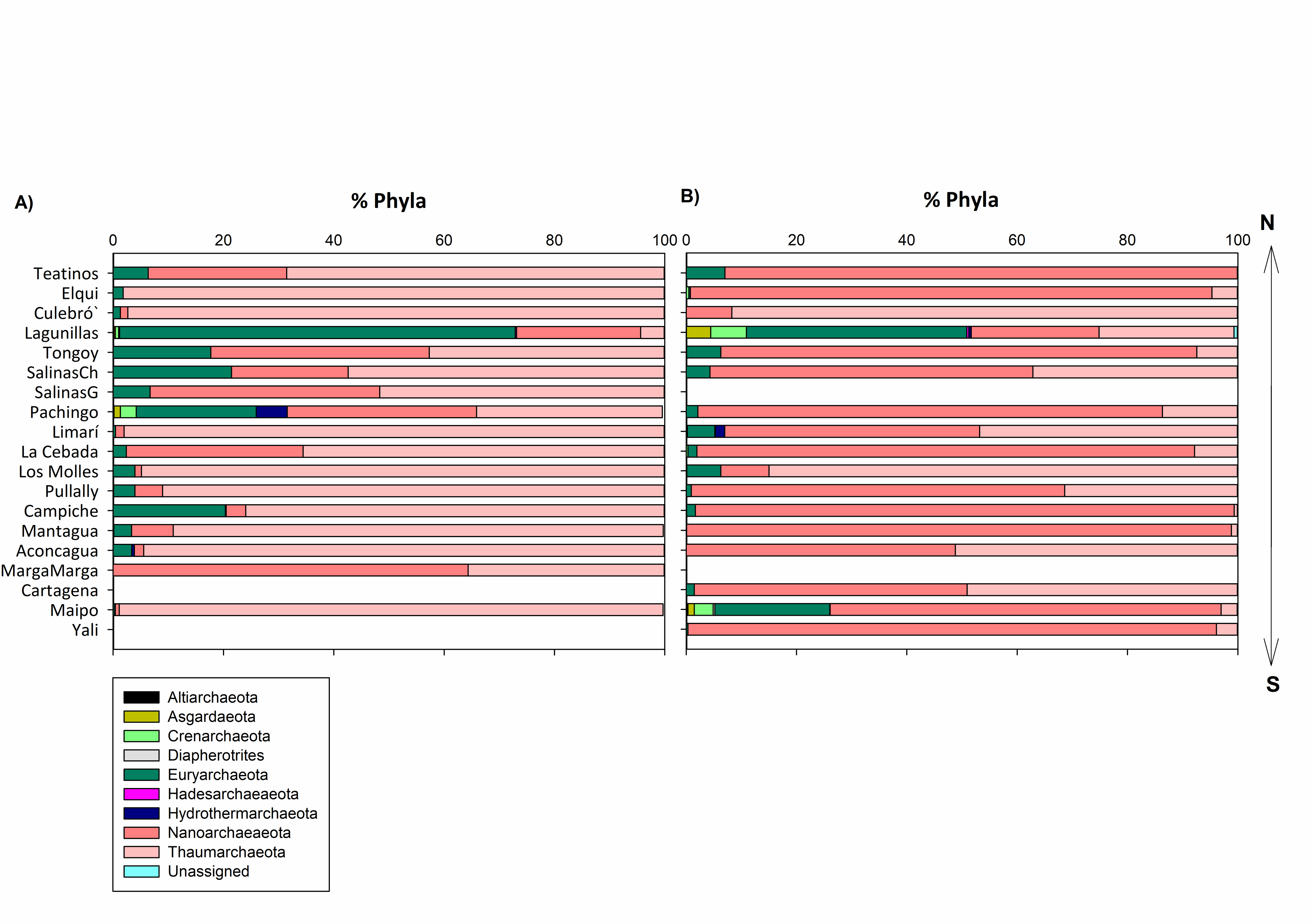

Supplement: S6 Fig — Benthic archaea phyla retrieved in winter (A) and summer (B). (TIF) [file pone.0271208.s013.tif]

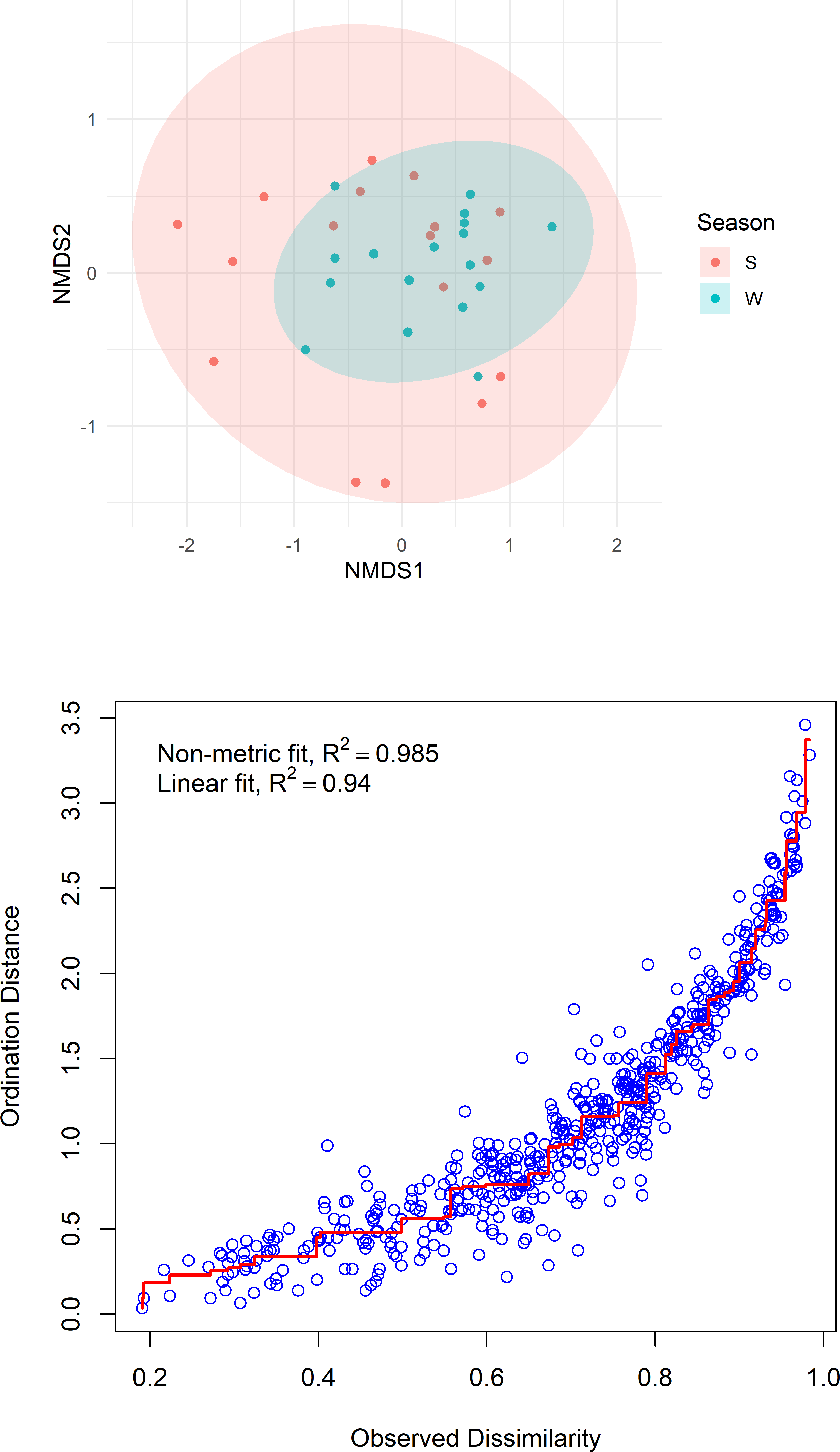

Supplement: S7 Fig — A) Non-metric multidimensional scaling (NMDS) analysis for selected functional groups (methane oxidizing bacteria, ammonia oxidizing bacteria and archaea, and methanogens). B) Linear fit associated with the NMDS analysis. (TIF) [file pone.0271208.s014.tif]
